# Overexpression of *VEGF* in dermal fibroblast cells accelerates the angiogenesis and wound healing function: in vitro and in vivo studies

**DOI:** 10.1038/s41598-022-23304-8

**Published:** 2022-11-02

**Authors:** Forough Shams, Hamideh Moravvej, Simzar Hosseinzadeh, Ebrahim Mostafavi, Hadi Bayat, Bahram Kazemi, Mojgan Bandehpour, Elnaz Rostami, Azam Rahimpour, Hamidreza Moosavian

**Affiliations:** 1 Department of Medical Biotechnology, School of Advanced Technologies in Medicine, Shahid Beheshti University of Medical Sciences, Tehran, Iran; 2 Skin Research Center, Shahid Beheshti University of Medical Sciences, Tehran, Iran; 3 Medical Nanotechnology and Tissue Engineering Research Center, Shahid Beheshti University of Medical Sciences, Tehran, Iran; 4 Department of Tissue Engineering and Applied Cell Sciences, School of Advanced Technologies in Medicine, Shahid Beheshti University of Medical Sciences, Tehran, Iran; 5 Stanford Cardiovascular Institute, Stanford University School of Medicine, Stanford, CA USA; 6 Department of Medicine, Stanford University School of Medicine, Stanford, CA USA; 7 Department of Molecular Genetics, Faculty of Biological Sciences, Tarbiat Modares University, Tehran, Iran; 8 Department of Animal Sciences and Biotechnology, Faculty of Life Sciences and Biotechnology, Shahid Beheshti University, Tehran, Iran; 9 Department of Clinical Pathology, Faculty of Veterinary Medicine, University of Tehran, Tehran, Iran

**Keywords:** Biomaterials, Gene therapy, Biotechnology

## Abstract

Fibroblasts are the main cells of connective tissue and have pivotal roles in the proliferative and maturation phases of wound healing. These cells can secrete various cytokines, growth factors, and collagen. Vascular endothelial growth factor (VEGF) is a unique factor in the migration process of fibroblast cells through induces wound healing cascade components such as angiogenesis, collagen deposition, and epithelialization. This study aimed to create VEGF_165_ overexpressing fibroblast cells to evaluate angiogenesis function in wound healing. In vitro, a novel recombinant expression vector, pcDNA3.1(-)-VEGF, was produced and transfected into the fibroblast cells. Following selecting fibroblast cells with hygromycin, recombinant cells were investigated in terms of VEGF expression by quantifying and qualifying methods. Mechanical, physical, and survival properties of polyurethane-cellulose acetate (PU-CA) scaffold were investigated. Finally*, *in vivo, the angiogenic potential was evaluated in four groups containing control, PU-CA, PU-CA with fibroblast cells, and VEGF-expressing cells on days 0, 2, 5, 12 and 15. Wound biopsies were harvested and the healing process was histopathologically evaluated on different days. qRT-PCR showed VEGF overexpression (sevenfold) in genetically-manipulated cells compared to fibroblast cells. Recombinant VEGF expression was also confirmed by western blotting. Manipulated fibroblast cells represented more angiogenesis than other groups on the second day after surgery, which was also confirmed by the antiCD31 antibody. The percentage of wound closure area on day 5 in genetically-manipulated Hu02 and Hu02 groups showed a significant reduction of wound area compared to other groups. These findings indicate that overexpression of *VEGF*_*165*_ in fibroblast cells results in enhanced angiogenesis and formation of granulated tissue in the early stage of the healing process, which can show its therapeutic potential in patients with impaired wound healing and also provide functional support for gene therapy.

## Introduction

Wound healing is a complex and dynamic process that is crucial to support the body against foreign pathogens and residue. Following skin injuries accelerated wound healing not only plays a pivotal role in preventing disease complications, such as secondary infections, but it is also important for the patient to return to normal as soon as possible and use the damaged organ. Also, in many patients who underwent flap reconstruction and tissue engineering, some techniques in accelerated wound healing can be crucial to reducing the risk of transplant rejection.

The natural wound-healing phase is a cascade of organized phenomena that appertains on multiple cells and mediators corresponding in a rather intricated time series after damage^1^. These processes consist of four distinct but overlapping stages; hemostasis, proliferative, inflammatory, and remodeling to regenerate and ameliorate the injured tissue^2^. Angiogenesis is critical in forming new vasculature to eliminate debris and prepare oxygen and nutrients for the metabolic active wound bed. Activated platelets during the hemostasis phase by releasing a host of factors (e.g., angiostatin, CXCL4) increase the primary inhibition of angiogenesis^3^. Many growth factors and cytokines (proangiogenic molecules) are secreted during the inflammatory process by lymphocytes, macrophages, and epithelial cells to recruit fibroblast cells into the injury area. In this way, the wound bed turns into an angiogenic sink that enhances endothelial cell migration, proliferation, and developing neovascularization of the wound bed^4^. The newly formed vasculature (vasculogenesis) developed from endothelial progenitor cells (EPC) is transferred to the swollen wound area and differentiated. Vasculogenesis is promoted via the employment and induction of differentiation by different angiogenic factors such as VEGF and stromal cell-derived factor 1 (SDF-1, CXCL12). Neovascularization of the wound bed and angiogenesis (granulation phase) are entirely progressing from day 4.

In the granulation phase, a multiplication increase in tissue vessels (about fivefold) occurs in the wound bed to provide the metabolic requirements of the cells regenerating the tissue^5^. In the wound healing process, the first fibroblasts appear at the end of the inflammatory stage and the beginning of the proliferative stage (24–48 h post-injury) at the injury site^6^. Chemoattractants such as platelet-derived growth factor (PDGF), interleukin-1 beta (IL-1β), and transforming growth factor-α (TNF-α), which are released from the temporary matrix by macrophages and platelets as part of the inflammatory reaction are critical in leading fibroblasts to the wound area.

Extracellular matrix (ECM) regulates and supports the activity and migration of the fibroblasts. Furthermore, this matrix signals and patronizes angiogenesis, granulation-tissue production, and epithelialization^7^. At this step, the granulation tissue is produced and affected by the newly generated vasculature. In this way, controlled angiogenesis which develops during the proliferative process of repair results in new blood vessel organization and vascular hyperpermeability, facilitating nutrient and oxygen transfer^8^. The application of angiogenic molecules, such as VEGF-A, known as vascular permeability factor, may enhance the permeability of capillaries. VEGF has five isoforms developing from alternative splicing of its mRNA, with chain lengths of 121, 145, 165, 189, and 206 amino acids. These five isoforms are VEGF-A (VEGF_165_), VEGF-B, VEGF-C, VEGF-D, and placental growth factor (PlGF), where VEGF_165_ is the most available and studied isoform. VEGF_165_ is produced by many cell types, such as endothelial cells, fibroblasts, macrophages, smooth muscle cells, neutrophils, and platelets, which are functionally involved in wound healing. This factor is unrivaled due to its effects on various components of the wound repair cascade, such as angiogenesis, collagen deposition, and epithelization^9,10^. The topical efficacy of VEGF and cell therapy has revolutionized the field of wound healing. VEGF alone or in combination therapy has been utilized to treat non-healing wounds^9^. Although purified growth factors and cell culture have been confirmed by the Food and Drug Administration (FDA) to improve non-healing wounds, sustained expression and a long half-life of the growth factors were not outstanding in this approaches^9,11,12^. There is some proof that the direct delivery of recombinant growth factors to the wound site could not be a practical therapeutic option, given the drawbacks of a short half-life of the growth factors, fast degradation, high cost, and unforeseeable harmful effects^13,14^. In vivo delivery of VEGF-A protein or mRNA for a shorter duration was less effective in repair. It has been suggested that sustained expression of VEGF-A during the initial angiogenic phase from particular cells can be more effective for optimal wound healing^15^. Gene delivery or genetic modification approaches assist researchers in developing more favorable particularity in cells and tissues. The recent advances in gene delivery systems include the progress of viral and non-viral gene delivery systems. Although the performance of transfecting host cells is approximately high with viral vectors compared to non-viral methods, the principal disadvantages of viral vectors are their cytotoxicity and immunogenicity^15^. Hence, chemical non-viral vectors such as liposomes that facilitate gene delivery are the most used in this field.

The gradual comprehension of the biological processes involved in wound healing has opened the entry for providing smart bio-constructs and modified cells that actively develop tissue regeneration through appropriately engineered regeneration platforms, scaffolds, and the efficient manipulation of cells. In order to stable delivery of manipulated cells in the wound bed, utilization of a matrix material or scaffold that can accommodate the delivery system is favorable. This study developed a polyurethane-cellulose acetate (PU-CA) scaffold that has provided regenerative potential in wounds. Up to now, a wide range of strategies has been performed to fabricate wound healing scaffolds, such as molding, extrusion, freeze-drying, and electrospinning. The electrospinning technique stays the most used among the viable candidates, owing to its low cost, simplicity, scalability, and flexibility^16^. PU-CA electrospun nanofibrous scaffolds provide a broad range of promising applications for wound dressing. Cellulose is one of the most plentiful polymers in nature and has received considerable attention because of its desirable properties. Cellulose acetate (CA) is one of the most prominent cellulose derivatives with desirable biocompatibility, non-toxicity, biodegradability, abundance, affordability, renewability, and versatility^17^. However, CA cannot be applied alone as a biomedical material owing to its low breaking stress, poor resistance, and strain. Therefore, polyurethane (PU) with both polyol as a soft segment and isocyanate as a hard segment has represented high efficacy in solidity and control of wound moisture. The wound dressings with PU compound have accelerated the healing rate due to their worthy mechanical properties and chemical attributes such as elasticity, tensile, strength, and oxygen permeability^18^. Since PU is a hydrophobic polymer, releasing fluid exudes from the wound surface is too hard. In contrast, CA, a highly hydrophilic polymer, could benefit water absorption and liquid transportation. Hence, the combination of PU and CA could decrease the limitation of both polymers. The electrospun PU-CA scaffold can be used as external support for cell proliferation and adhesion, which improve tissue regeneration and reorganization^19^.

**Figure 1:**
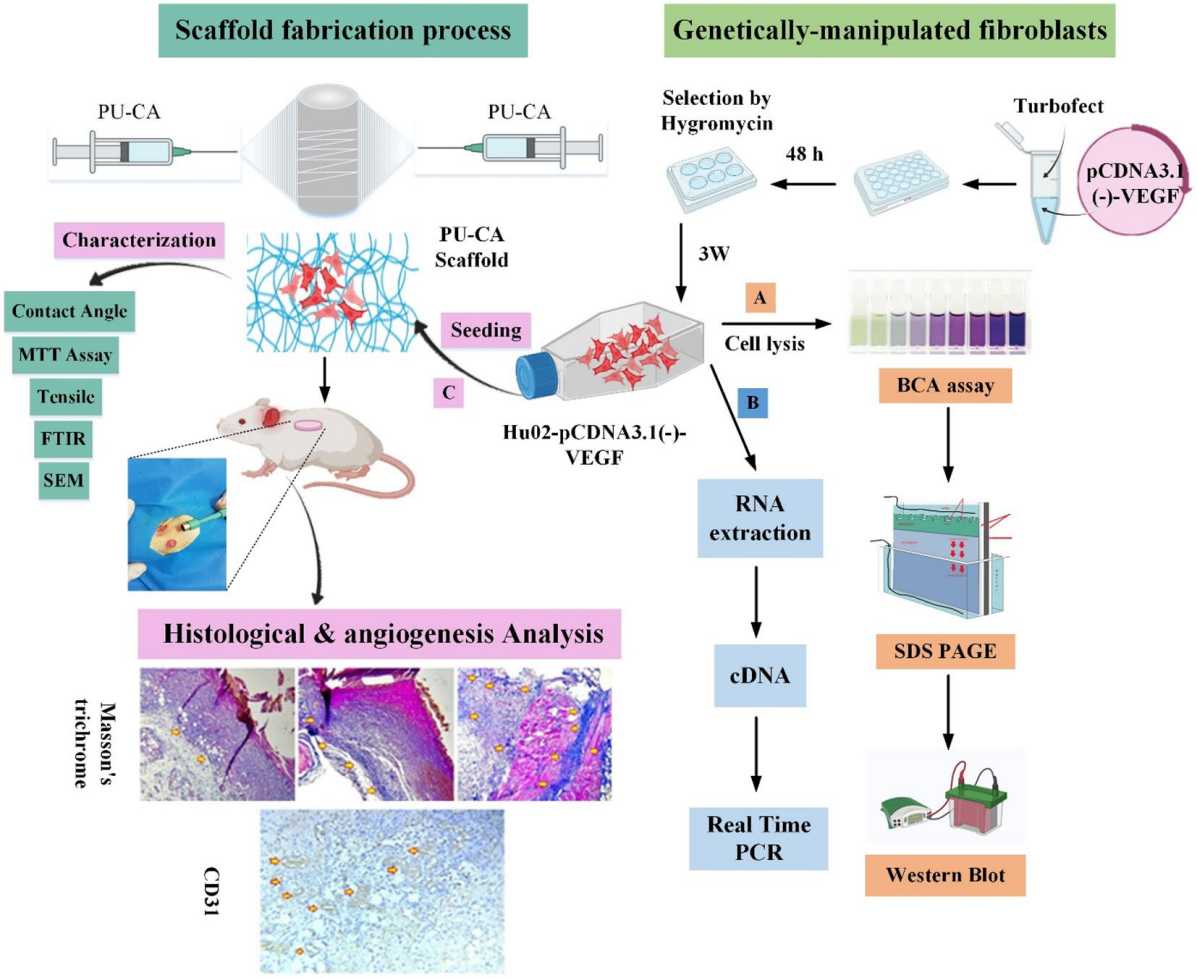
Schematic illustration of in vitro and in vivo studies (Graphical Abstract). The genetically manipulated procedure was begun by constructing pcDNA3.1(-)-VEGF recombinant expression vector and then transfected into the fibroblast cells. Following the selection of fibroblast cells with hygromycin, recombinant cells were investigated in terms of VEGF expression by (**A**) real-time PCR and (**B**) western blotting methods. After the scaffold fabrication process, the mechanical, physical, and survival properties of polyurethane-cellulose acetate (PU-CA) scaffold were investigated. (**C**) Manipulated fibroblast cells were seeded on a PU-CA scaffold to assess the angiogenic potential in four groups containing control, PU-CA, PU-CA with fibroblast cells, and VEGF-expressing cells on different days. The healing process and angiogenesis were histopathologically evaluated by H&E, Masson’s trichrome staining, and the IHC test via the CD31 marker.

Given the importance of VEGF during wound healing, the current study set out to enhance the innate capacity of fibroblasts by transfection of the *VEGF*_*165*_ gene for overexpression. In terms of VEGF expression, manipulated cells were investigated by quantifying and qualifying methods. Finally, angiogenesis function was investigated in fibroblasts and manipulated cells on PU-CA scaffold during tissue regeneration following third-grade skin wounds in rats. The graphical abstract of the study is represented in Fig. 1.

## Results

### Recombinant expression vector construction

**Figure 2:**
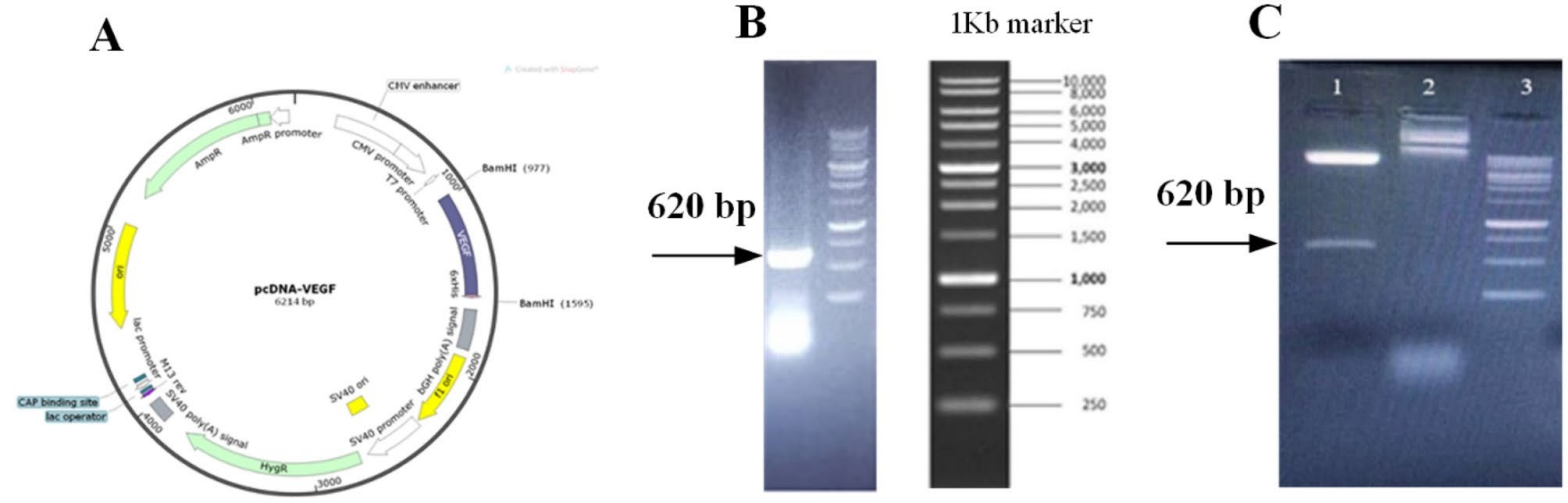
Schematic representation of the expression vector containing targeted gene used in this study and confirmation of *VEGF* gene by PCR and final approval of cloning by *BamHI* restriction enzyme. (**A**) pcDNA 3.1 (-) expression vector containing VEGF fragment downstream of the CMV promoter. (**B**) Agarose gel electrophoresis revealed that the PCR amplified target gene fragment was in the expected position, 620 bp of VEGF. (**C**) Cloning confirmation was done from the intermediate vector in the expression vector via *BamHI* restriction enzyme. Column 1: pcDNA 3.1 (-) containing VEGF vector cut with *BamHI* enzyme that cut fragment is 620 bp Column 2: The uncut pcDNA 3.1 (-) containing VEGF vector. Column 3: 1 Kb molecular weight size marker (Thermos Fisher Scientifics, USA).

This study generated an expression vector where *VEGF*_165_ was cloned in pcDNA 3.1 (-) Hygro vector containing a human CMV promoter. Figure 2A indicates the schema of the final pcDNA 3.1(-)-VEGF vector. Following PCR amplification (Fig. 2B, Supplementary Fig. 1A) and restriction digestion (Fig. 2C, Supplementary Fig. 1B), the target gene fragments were observed in agarose gel electrophoresis.

### Development of stable cell pools and analysis of VEGF overexpression in vitro

**Figure 3:**
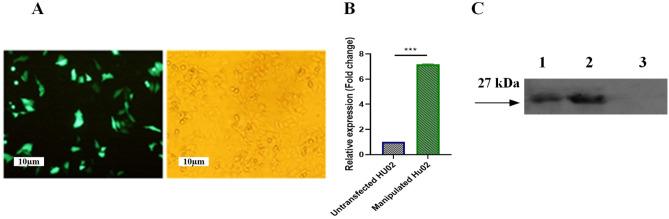
Transfection efficiency and investigating overexpression of VEGF gene in manipulated Hu02. (**A**) Fluorescent image of transfected Hu02 cells expressing EGFP (green fluorescence) as a transfection control. (**B**) Fold change of VEGF mRNA expression in manipulated Hu02 compared to untransfected Hu02 was quantified using qRT-PCR. B-actin was used as an internal control. (**C**) Western blotting analysis of VEGF overexpression among (1) control, (2) transfected, and (3) untransfected groups. (***P ≤ 0.001).

The transfection of recombinant pcDNA 3.1 (-) vector containing VEGF and EGFP plasmid into the fibroblast cells was successfully performed using TurboFect Transfection Reagent. The transfection efficiency was evaluated by the EGFP expression in the pEGFP-transfected cells (Fig. 3A). Then, the pools of fibroblasts harboring pcDNA 3.1 (-) vector containing VEGF were developed by selecting transfected cells in a hygromycin-containing medium. The VEGF-overexpressing pools were collected following three weeks of selection. The expression of *VEGF* in manipulated Hu02 was confirmed through qRT-PCR and western blot analysis. qPCR analysis demonstrated a significant change in the mRNA levels of the *VEGF* gene expression in genetically-modified Hu02 by 7 times in comparison to untransfected Hu02 (***p-value < 0.001 (Fig. 3B). A significant change was demonstrated through qPCR analysis in the VEGF expression level in genetically-modified Hu02 by 7 times in comparison to untransfected Hu02 (***p-value < 0.001) (Fig. 3B). Western blot analysis approved the VEGF overexpression in recombinant Hu02 compared to the Hu02 group (Fig. 3C, Supplementary Fig. 1C). As expected, in VEGF engineered cells, overexpression of VEGF was validated by His-Tag with a relative molecular weight of ~ 27 kDa, while no expression was observed in Hu02 cells.

### Morphological observation of scaffold

**Figure 4:**
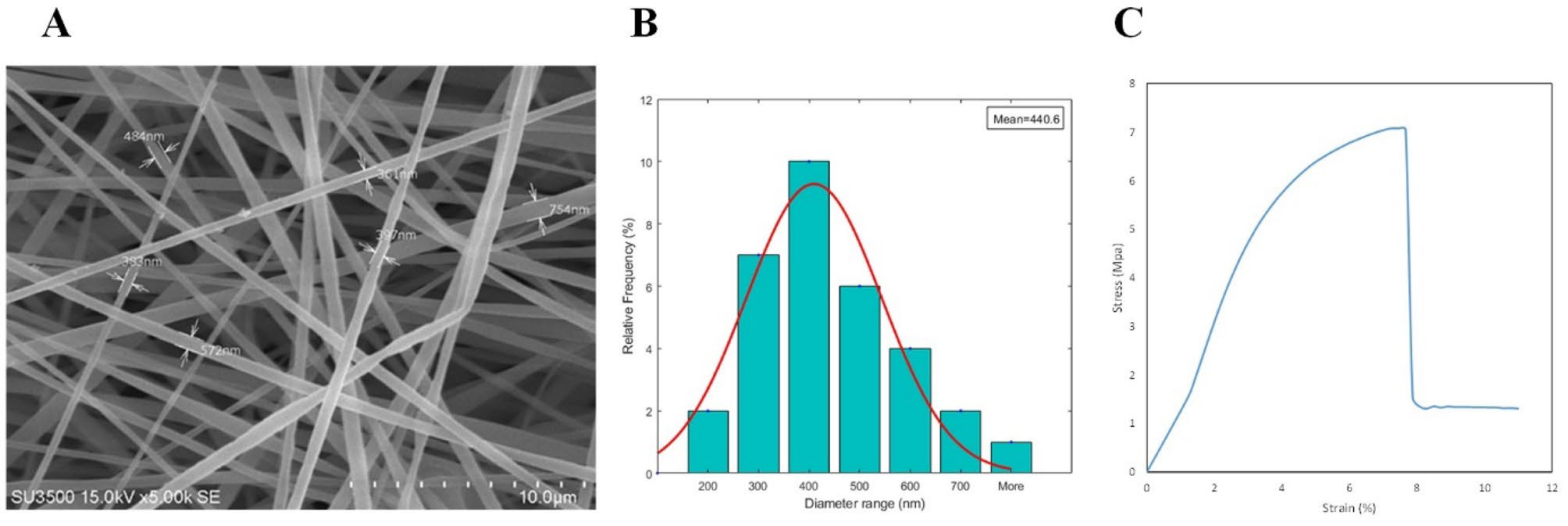
(**A**) Morphology, (**B**) diameter distribution and (**C**) representative stress–strain curve of PU-CA scaffold.

The morphology of fabricated fibers was represented using SEM imaging (Fig. 4A). Also, the mean diameter and diameter distribution of the scaffold were investigated. The SEM demonstrated that the fabricated PU-CA fibers are uniform, regular, willow-free, and deformable. The image analysis utilizing Image J software revealed that the mean diameter of the PU-CA scaffold was 623.66 nm. The diameter distribution of PU-CA fibers was as follows: 28.1% of fibers were less than 300 nm diameter, 62% ranged between 300 and 600 nm diameter, and 9.3% were more than 600 nm diameter (Fig. 4B).

### Mechanical property

Evaluation of mechanical properties is necessary for biomedical applications. The mechanical parameters of the fibers were investigated by the tensile strength (TS) procedure according to a standard test. The mechanical parameters of PU-CA, such as ultimate tensile strength, strain at break, and young modulus, were assessed by acquiring the stress–strain curve of the scaffold (Fig. 4C). The results showed the PU-CA's tensile strength (%), young modulus, and elongation at break (MPa) were 14.51529, 1.59, and 7.075214, respectively. The elongation at the breakpoint is the strain obtained by the scaffold at the ultimate stress. Young modulus was measured as the slope of the linear area through the stress–strain curve. The strain value demonstrates the elongation of PU-CA compared to its initial length. The ultimate strength was also measured as the maximum stress executed to the scaffold that, in this study, was the point at which the scaffold had been teared. According to the results, the presence of PU and CA showed excellent elasticity and strength, respectively.

### Contact angle measurement

**Figure 5:**
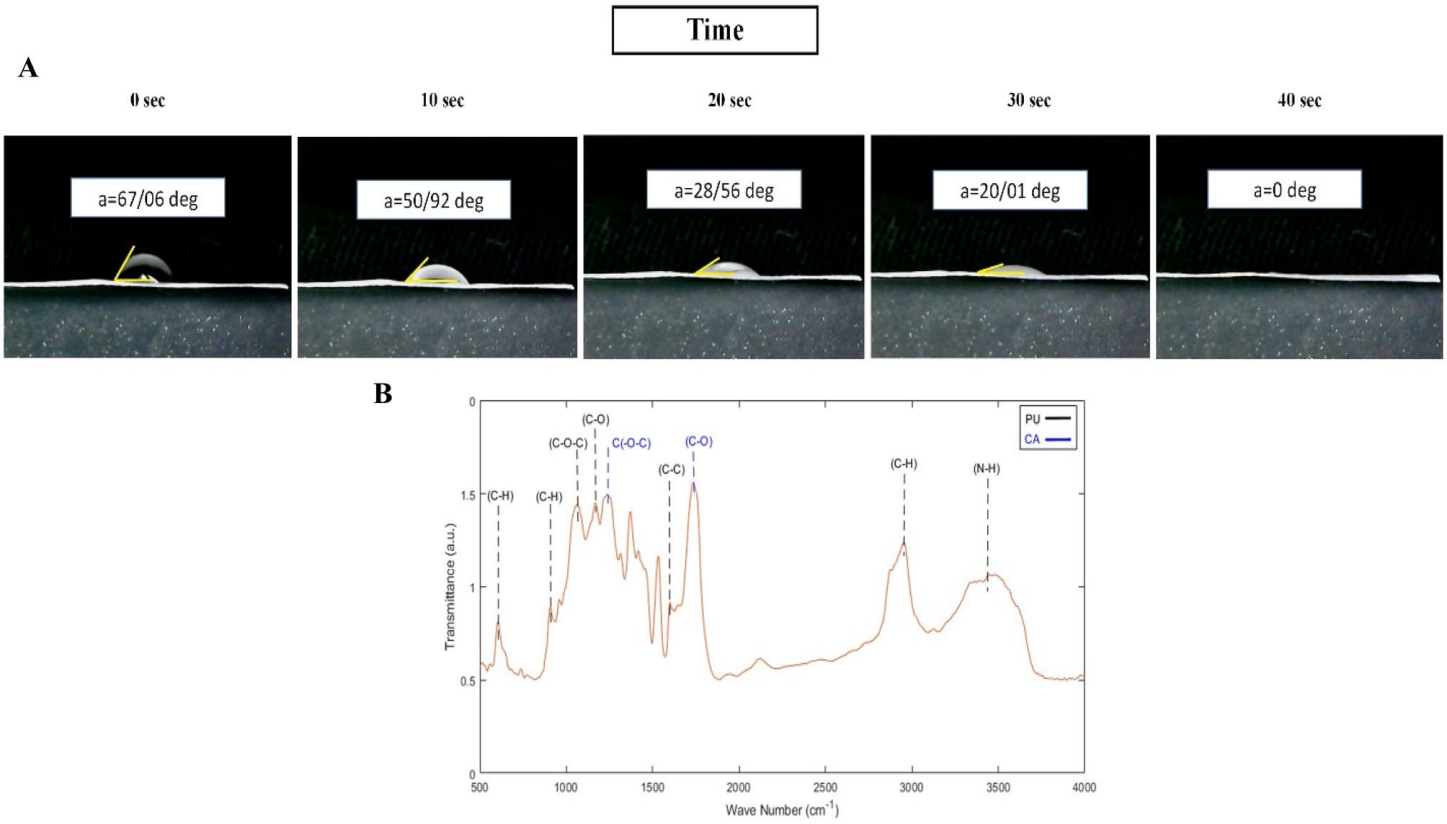
(**A**) Contact angle measurements of PU-CA scaffold and (B) FTIR spectra of PU-CA scaffold that black and blue dashed line shows PU and CA peaks, respectively.

The contact angle test is an appropriate technique to evaluate the surface wettability of the scaffold. Figure 5A showed that the PU-CA scaffold was classified as a hydrophilic surface after 40 s, a positive point for cell attachment. The contact angle of the PU-CA scaffold was 67.06°, which showed hydrophilic properties and made suitable conditions for cell attachment in the scaffold. A contact angle under 90° demonstrated the hydrophilicity characteristics of the scaffold.

### FTIR analysis

FTIR results determined the functional groups, structures, and molecular components of the PU-CA scaffold (Fig. 5B). The spectroscopy of the scaffold showed the characteristic peaks of PU at 3,444, 2939, 1597,1110, 1057,905, and 620 wavenumbers (ν) corresponding to NH, CH2, C2H4, C–O, C–O, C–O–C, C–H and benzene ring^20^. The adsorption peaks of CA were at 1235 cm^−1^ and 1743 cm^−1^ representing ν _C(-O-C)_ and ν _(C-O)_^21^.

### Cell viability assay

**Figure 6:**
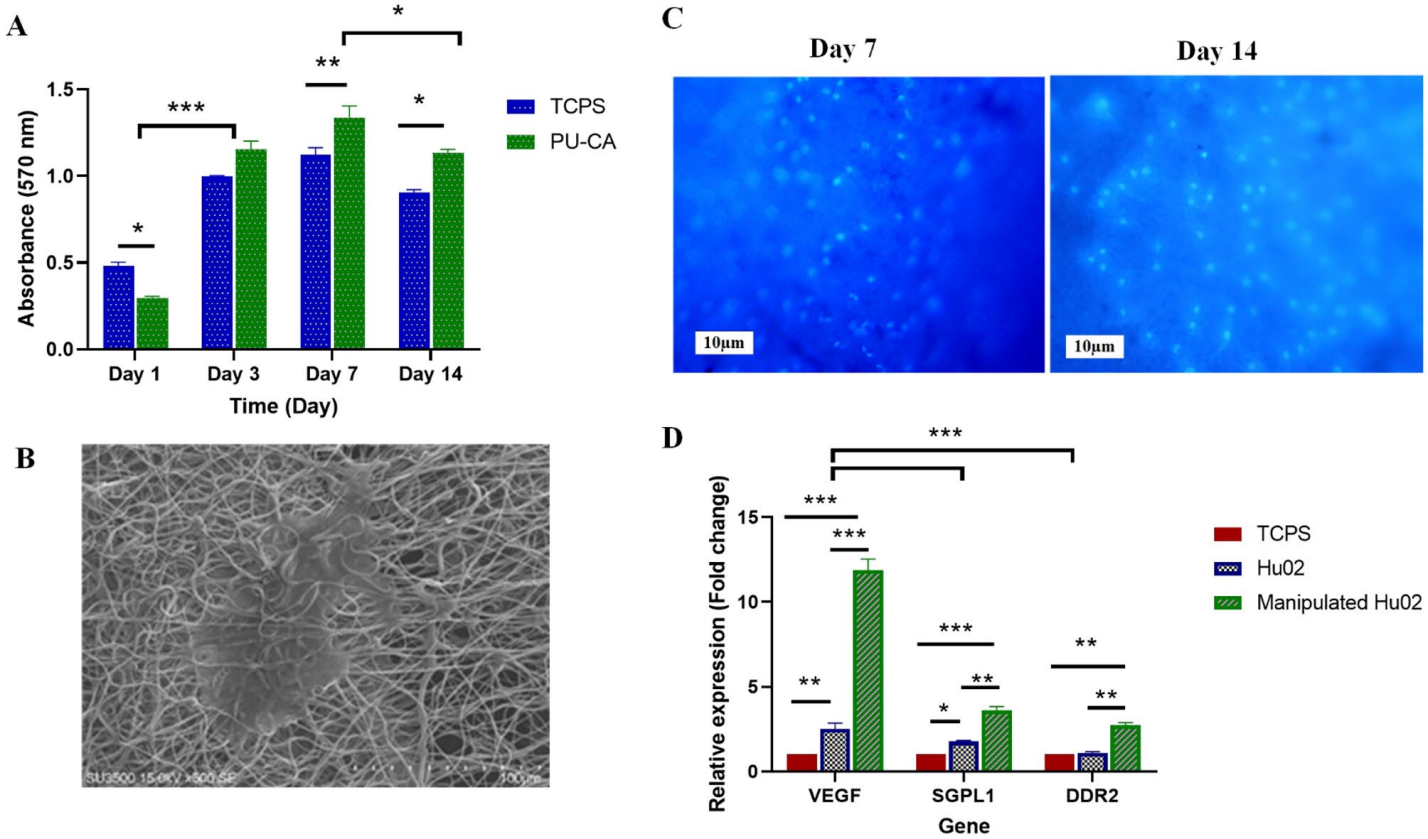
(**A**) Viability of fibroblasts seeded on scaffold and TCPS as control during the 14 days, (**B**) SEM images of fibroblasts on the surface of the PU-CA scaffold 3 days after cell culture. (**C**) DAPI staining after culture of cells on days 7 and 14. (**D**) *VEGF*, *DDR2*, and *SGPL1* gene expression at mRNA level. Three days after cell seeding on scaffold and TCPS the cells of each group were tested by Real-Time PCR. Manipulated cells showed more expression in certain genes compared to Hu02 and TCPS groups. (*P ≤ 0.05, **P ≤ 0.01, ***P ≤ 0.001).

The viability and cytocompatibility of the scaffold were investigated by the MTT method as a colorimetric assay at 570 nm. The optical density of the scaffold was evaluated and the absorbance values (standard and mean deviation) were measured after 1, 3, 7 and 14 days (Fig. 6A). The highest proliferation rate of fibroblasts on the scaffold was represented on days 3 and 7 compared to the TCPS group. On day one, the TCPS group had more active cells in comparison to the scaffold (*P* ≤ 0.05). The PU-CA and TCPS group showed a steady increase in cell number until day 7, while the number of active cells decreased on day 14. Moreover, the survival rate of cell viability in the scaffold group was higher than in the TCPS group on day 14.

### Cell attachment studies

Cell attachment to the scaffold was assessed by SEM and DAPI tests. SEM image (Fig. 6B) showed that manipulated cells adhered well to the scaffold and its surface developed a proper microenvironment for cell–cell and cell–matrix connection after 3 days. DAPI staining images were taken through fluorescence microscopy on days 7 and 14 (Fig. 6C). Images represented the attachment of fibroblast cells with a healthy nucleus to the scaffold observed in blue color with a dark field.

### Gene expression in the cells seeded on the scaffold

Relative expression of *VEGF*, *DDR2,* and *SGPL2* genes was evaluated three days after cell seeding to indicate the angiogenesis, migration, and proliferation potential of the manipulated cells on the scaffold. qRT-PCR analysis demonstrated a significant increment in selected genes at mRNA level in manipulated fibroblasts seeded on the scaffold in comparison to Hu02 and TCPS group (Fig. 6D). In manipulated group, the expression of the *VEGF*, *DDR2*, and *SGPL1* genes at the mRNA level was about 5, 2.5, and 2.1-fold higher than the Hu02 group, respectively (*P* ≤ 0.001). Also, a significant increase was represented in the expression level of the *VEGF* and *SGPL1* genes (2 and 1.7-fold) in Hu02 cells compared to the TCPS group (*P* ≤ 0.01, 0.05). The *VEGF* and* SGPL2* gene expression level in both cells increased significantly compared to TCPS as the control.

### In vivo wound healing analyzes

**Figure 7:**
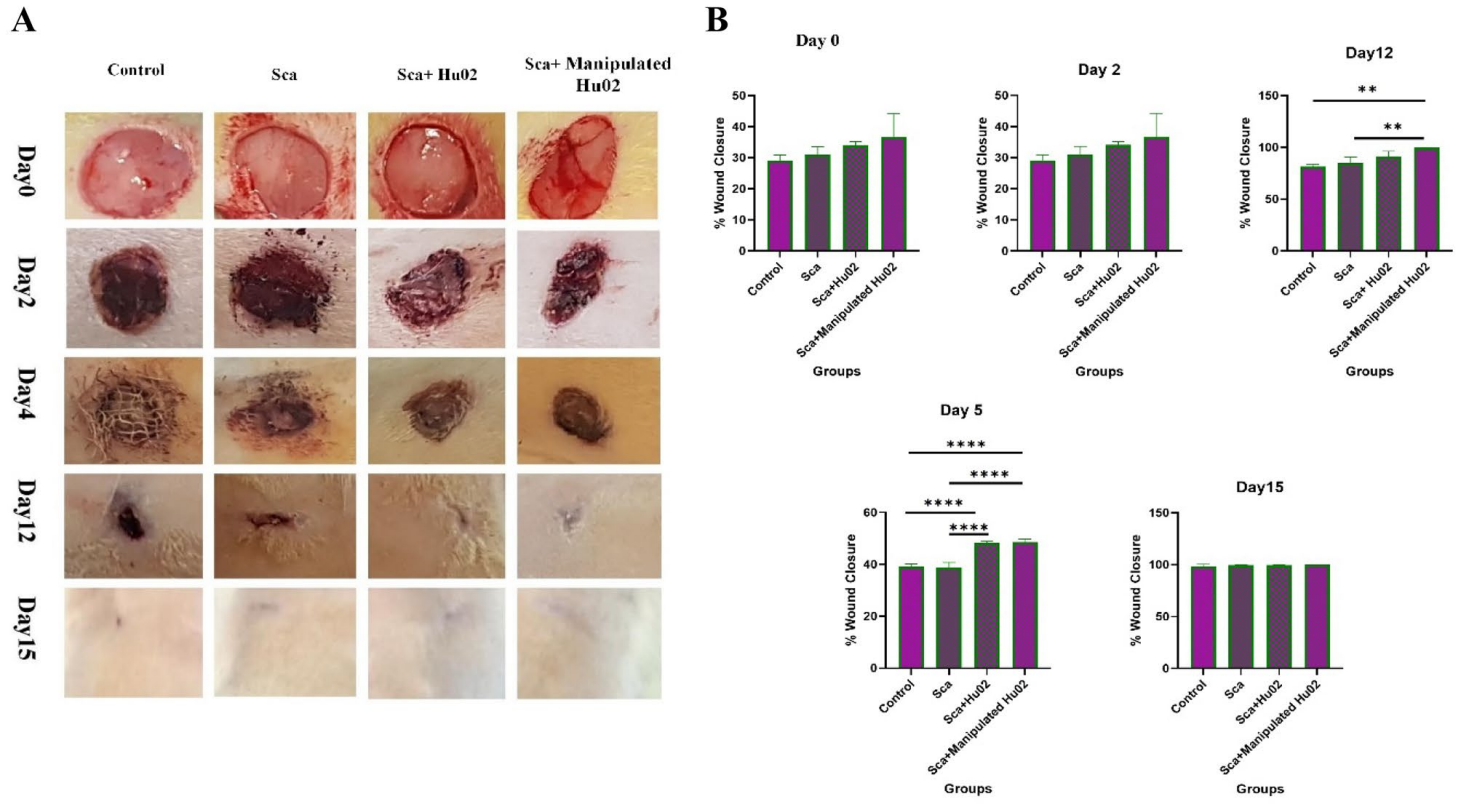
(**A**) Macroscopic pictures of the healing process at definite time points (at days 0, 2, 5, 12, and 15 after wound creation) show wound closure progression in different groups (Ctrl, Sca, Sca + Hu02, and Sca + Manipulated Hu02). (**B**) The level of wound closure progression as a percentage of the remaining wound area at definite time points. (**p < 0.01, ****p < 0.0001).

The efficacy of manipulated cells and Hu02 on the PU-CA wound dressing was evaluated on the PU-CA and control group in the Wistar rats’ full-thickness wound model. All specimens were situated in the wound area and the wound healing process was monitored for 15 days. No significant changes were shown in the bodyweight of the experimental groups. During the experiment, rats survived and were sacrificed on certain days for histopathological examinations. The remaining wound area’s percentage was measured based on wound diameters at the definite time points (0, 2, 5, 12 and 15 days after the operation). Figure 7A shows representative wound images obtained for each treatment group on days post-wounding. Also, Fig. 7B demonstrates the percentages of the wound closure area. On day 2, no significant variations were detected in the wound area of rats treated with manipulated Hu02 and Hu02 compared with two other groups (Scf and Control). In contrast, on day 5, both groups treated with VEGF-expressing Hu02 and Hu02 showed a significantly greater wound area reduction comparing the scaffold (*p* < 0.0001) and control group (*p* < 0.0001). On day 12, the group treated with genetically-manipulated Hu02 exhibited significantly greater wound closure with respect to the scaffold (*p* < 0.01) and control group (*p* < 0.01). On day 15, the mean size of the wound area in different groups of rats was not significant.

### Histological analysis

**Figure 8:**
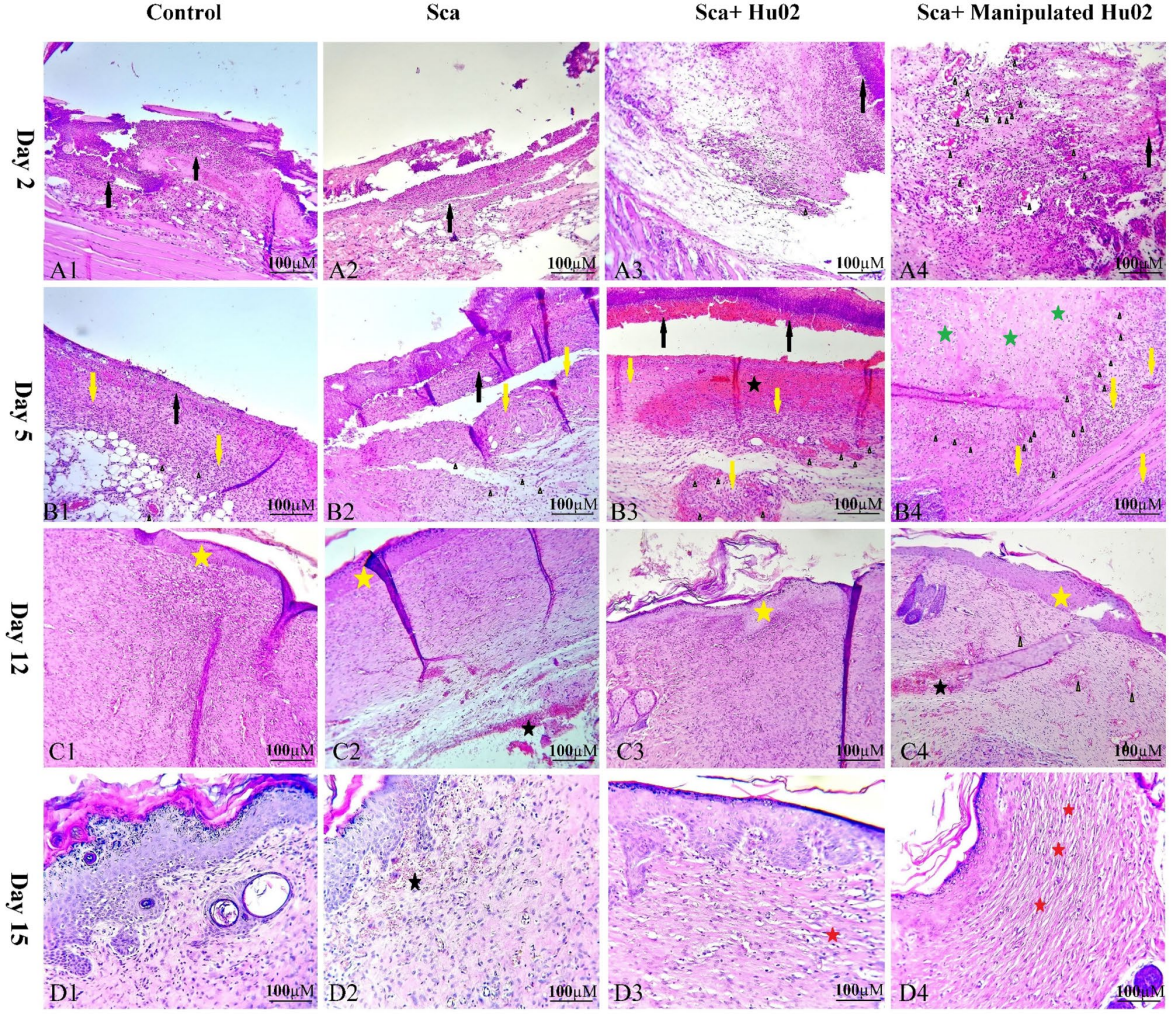
H&E stained microscopic sections of wounded tissue on different days after surgery for Ctrl, Sca, Sca + Hu02, and Sca + Manipulated Hu02 groups. (**A1**)–(**A4**): Day 2 after surgery. (**A1**) and (**A2**): Tissue necrosis with high neutrophil infiltration (black arrow) and no angiogenesis. (**A3**): Tissue necrosis with high neutrophil infiltration (black arrow) and with mild angiogenesis as few formation of new blood vessels (arrow head). (**A4**): Tissue necrosis with mild neutrophil infiltration (black arrow) and remarkable angiogenesis (arrow head). (**B1**)–(**B4**): Day 5 after surgery. (**B1**) and (**B2**): Tissue necrosis with neutrophil infiltration (black arrow), mild angiogenesis (arrow head), mild fibroproliferation, and new connective tissue formation (yellow arrow). (**B3**): Tissue necrosis with neutrophil infiltration (black arrow), moderate angiogenesis (arrow head), hemorrhage (black star), high fibroproliferation, and new connective tissue formation (yellow arrow). (**B4**): Remarkable angiogenesis (arrow head), moderate fibroproliferation (yellow arrow), and new connective tissue formation and collagen deposition (green stars). (**C1**)–(**C4**): Day 12 after surgery. (**C1**): Reepithelialization (yellow star) and increased connective tissue. (**C2**): Reepithelialization (yellow star), increased connective tissue, and some area of hemorrhage (black star). (**C3**): Reepithelialization (yellow star) and increased connective tissue. (**C4**): Reepithelialization (yellow star), increased connective tissue, and some area of hemorrhage (black star). Granulation tissue and angiogenesis (arrow head) were present in some areas. (**D1**)–(**D4**): Day15 after surgery. (**D1**): Reduction of fibroproliferation. (**D2**): Reduction of fibroproliferation and the presence of subepidemal hemorrhage (black star) in some area. (**D3**): Reduction of fibroproliferation and beginning of scar tissue formation (red star). (**D4**): Reduction of fibroproliferation and remarkable scar tissue formation as large parallel bundles (red star).

**Figure 9:**
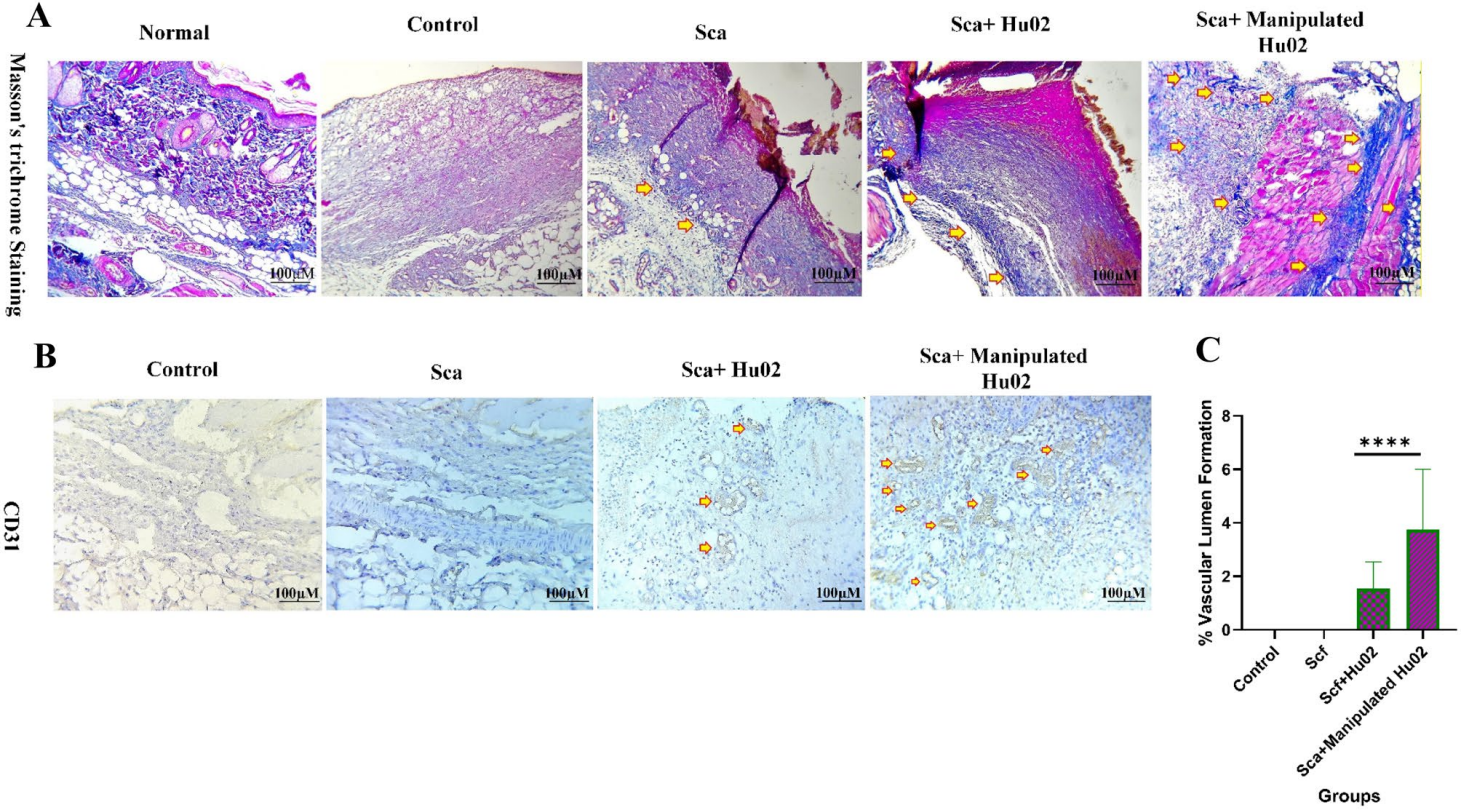
(**A**) MT staining of the tissue biopsies on the 5th day of wound healing. The highest amount of collagen bundles regeneration (arrow) was in Manipulated Hu02, Hu02, and Scf groups, respectively. (**B**) Comparison of different treatment groups for angiogenesis by anti-CD31 on the 2nd day of wound healing. No angiogenesis was seen in the control and Scf groups, while few and remarkable angiogeneses (arrow) were detected in the Hu02 and Manipulated Hu02 groups, respectively. (**C**) Quantitative evaluation of vascular lumen formation by CD31 immunostaining in Control, Sca, Sca + Hu02, and Manipulated Hu02; ****p < 0.0001.

Figure 8 illustrates histological examinations of skin specimens from the wound area in four groups on the 2nd, 5th, 10th and 15th days after wound creation. On the second day, the VEGF-expressing Hu02 (VEGF-Hu02) demonstrated remarkable angiogenesis with moderate neutrophil infiltration, while the other groups showed low or no angiogenesis with high neutrophil infiltration (Supplementary Fig. 2). The VEGF-Hu02 showed remarkable angiogenesis, moderate fibroproliferation, and new connective tissue formation on the fifth day. In contrast, the other groups showed mild or moderate angiogenesis, mild or high fibroproliferation, and new connective tissue formation. On the twelfth day, all groups represented re-epithelialization and increased connective tissue. In the VEGF- Hu02 group, granular tissue and angiogenesis were shown in some areas, while no angiogenesis was shown in others. On the fifteenth day, the VEGF- Hu02 group demonstrated remarkable scar tissue formation, while scar tissue formation began in the fibroblast group and was not seen in other groups. Masson’s trichrome was used to stain the specimens on day 5 for evaluating collagen deposition. Accumulation of collagen fibers and collagen deposition were detected in all specimens except the control group (Fig. 9A). This staining showed more accumulation of collagen organized in the rat treated with the manipulated group compared to other groups.

Three critical processes, including inflammation, proliferation, and maturation, were evaluated in the tissue repair process. (i) On the second day after wound induction, inflammation was seen in all studied groups and continued for a few days. The severity of inflammation on the fifth day was lower in the Sca + Manipulated Hu02 group compared to other groups. (ii) In evaluating the proliferation stage, which includes the proliferation of fibroblasts, collagen deposition, angiogenesis, formation of granulation tissue, and new epithelial formation, the first evidence of angiogenesis was seen on the second day in the Sca + Manipulated Hu02 group. However, angiogenesis was not found in the control and scaffold groups, and angiogenesis in the Sca + Hu02 group was very low on the second day. (iii) On the 15th day, the first evidence of collagen maturation was seen in the form of collagen bundles in the sub-epithelial connective tissue in the Sca + Manipulated Hu02 group. In the Sca + Hu02 group, the formation of collagen bundles was seen in a scattered and partial manner, and it was not seen in the other groups.

Furthermore, the process of connective tissue formation and collagen deposition in the Sca + Manipulated Hu02 group was faster than in other groups, so on the fifth day after wound induction, significant connective tissue formation and intense fibroblastic proliferation were seen in the Sca + Manipulated Hu02 group, and the intensity of inflammation was reduced. On the fifth day, different degrees of inflammation, angiogenesis, and mild fibroblastic proliferation were seen in other groups.

### Evaluation of angiogenesis formation

CD31 immunostaining was performed on day 2 to evaluate early angiogenesis (Fig. 9B). This protein shows a particular marker for endothelial cells. Two groups of third-grade skin wounds represented positive staining. CD31 marker can be largely represented in the wound site of rats treated by VEGF- Hu02 group, while lower angiogenesis represented on rats treated by the Hu02 group at 2 days post-injury. The vascular and total areas were measured using Digimizer image analysis software (version 5.7.2). Using the images acquired from CD31 staining, the tissue samples were evaluated for the percentage of vascular lumen formation (Fig. 9C). The VEGF- Hu02 group showed a two and four-fold increase in vascular lumen formation (%) in comparison to the Hu02 group and two other groups (Scf & Control), respectively.

## Discussion

The current study found that forming new blood vessels, which mediates the transport of oxygen, nutrients, circulating cells, and growth factors, is a fundamental process for skin wound healing and tissue regeneration^22^. VEGF has recently attracted consideration due to increased angiogenesis and promoted vascular network expansion in tissue engineering. Indeed, VEGF is the key molecular target of angiogenesis expansion in regenerative skin medicine. The essential role of VEGF in repairing tissue has been recognized through local VEGF or in combination with cell therapy after inducing injury. Although the FDA has approved both purified VEGF and fibroblasts to accelerate closure of non-healing wounds, sustained expression and a long half-life of the growth factors were not outstanding in these approaches^9^. The gene delivery procedure is a potential substitution for the protein delivery approach to attain a therapeutic concentration of growth factors. This approach also provides the regulated and sustained expression of the *VEGF* gene with authentic post-translational modifications at the wound site^11^. Furthermore, through cell-based therapy such as fibroblasts and/or keratinocyte cells alone or in combination with membrane and skin grafts, wound healing accelerates^23^. In this line, most studies have used fibroblasts to treat a full-thickness wound^24,25^. However, the advantageous effects of cell therapy may be restricted owing to the rough environment of wounded sites, such as local ischemia, oxidative stress, and inflammation. With the development of gene therapy, obstacles such as a short half-life of the growth factors, fast degradation, high cost, and the rough environment of wounded sites can be eliminated by supporting cells against the obstacles during cell delivery and the harsh environment of the wound area. In most studies, overexpression of the VEGF was utilized for heart failure^26–28^. For instance, Augustin et al. have also investigated the effect of manipulated rat's MSCs to overexpress the VEGF gene through a lentiviral vector in acute myocardial infarction. They reported VEGF overexpression significantly decreased MSC apoptosis, increased VEGF secretion under hypoxic states, and enhanced capillary density in the infarct border zone^26^. However, overexpressing VEGF in the wound healing process was also observed in different cells through various gene transfer methods.

In the present study, overexpressing *VEGF* in the fibroblasts was used to evaluate the effects of *VEGF* upregulation in wound healing. Based on the literature, overexpression of *VEGF* and* BMP‑6* in bone marrow mesenchymal stem cells (BMSCs) enhanced bone formation and blood vessels, which develops theoretical patronage for gene therapy^29^. Similarly, Ni et al. indicated an in vitro model of the co‑overexpression of *Bcl‑2* and *VEGF* in MSCs. This genetic modification showed more rapid proliferation, a higher expression level of the target genes, reduced autophagy, decreased apoptosis, and increased paracrine effect^30^.

ECM includes nanostructural protein strands, which supply a three-dimensional matrix for attachment, differentiation, and proliferation of cells in human skin. A desirable scaffold should provide the characteristics of the ECM, i.e., three-dimensionality, nanostructure size, and porosity. Esmaeili et al. fabricated PU-CA and PU-CA-rGO nanofibers as the wound dressing materials and reported the nanofiber's diameters were 678 ± 235 and 254 ± 79 nm, respectively. They observed highly porous and nanostructure properties in PU-CA-based scaffolds, representing the high potential for being used in wound dressing^18^. In another study, Unnithan et al. demonstrated the fabrication of smooth and randomly oriented PU–CA–zein nanocomposite with a diameter of 550 nm^31^. Acquired nanofibers with various diameters in different studies can be related to added components like graphene, etc., and the different electrospinning parameters, such as distance of the nozzle to the collector, feeding rate, and applied voltage^32^.

The results showed that incorporating polyurethane with cellulose acetate improved the mechanical feature and increased the tensile strength. In this regard, Unnithan et al. reported that pure PU presented excellent elasticity compared to PU–CA–zein-drug nanocomposite^31^. Also, increasing young modulus enhances hardness approved in nanocomposite compared to pristine PU fibers. In this way, the presence of pristine PU enhances tensile strength while decreasing the strength of the scaffold. Based on our previous study, we obtained a reasonable equilibrium between hardness and flexibility by differentiating CA and PU^17^. Flexible and soft nanofiber scaffolds can be easily used for replacement due to easy handling and separation from the wound sites. Hence, the present scaffold contains enough attributes to be used as a wound dressing material.

The contact angle of the present scaffold indicated appropriate hydrophilicity to absorb exudates and sustain the moisture of the wound bed. Although a more hydrophilic nanofiber surface will benefit cell adhesion, it results in poor fiber stability. The presence of CA within the PU-CA scaffold due to its hydrophilic nature resulted in the increased wettability of electrospun fibers. Therefore, an optimized amount of PU could also be assisted in keeping fiber stability and surface wettability. The hydrophilic attributes of the scaffold assisted cell attachment to wound dressing and decreased wound closure and the healing process^17,33^.

The FTIR findings confirmed the successful incorporation of PU and CA by the electrospinning method. When the PU- CA peaks were compared with the pristine PU in other studies, the peak intensities were decreased with CA content. This phenomenon was due to the formation of hydrogen bonds among the ingredients. The inter-hydrogen bonds created between two different macromolecules were more robust than those organized between the molecules of the same polymer^17,31^.

MTT results demonstrated enhancement in the number of active cells on days 1, 3, and 7 in the TCPS group, while decreased cell viability was shown on day 7 after seeding cells. The cell proliferation on the scaffold with reasonable speed was observed until day 14. Hence, the fabricated scaffold had appropriate biocompatibility with no toxicity for fibroblast cells, as indicated by other reports^18,34^.

The findings of the SEM images about cell proliferation and attachment on the electrospun scaffold were approved with DAPI staining. The hydrophilic essence of the PU-CA scaffold was the possible reason for appropriate adhesive behavior and proliferation of the cells. Moreover, the scaffold's porous structure plays a remarkable role in the proliferation and adhesion of the VEGF-Hu02 cells^31^. Based on data, the cell spreading and cell attachment in the nanofiber matrix can be clearly corroborated.

In addition to VEGF overexpression, VEGF-Hu02 cells showed a remarkable difference in the expression of migration markers compared to the Hu02 group. As mentioned in many different research studies, fibroblasts accelerate and improve wound repair by secreting ECM and other growth factors; thus, fibroblast manipulation directly affects the expression level of its genes^25^. For instance, Wang et al. evaluated the effects of adipose-derived stem cell extracellular vesicles (ADSCs-hEVs) on fibroblast cells. They showed that the expression levels of the *MMP1, VEGF*, *TGF-β*, and *EGF* genes were significantly increased in qRT-PCR analysis^35^. In another study, Salafutdinov et al. evaluated the efficiency of human umbilical cord blood mononuclear cells (hUCB-MC) transfection with the pBudVEGF-FGF2 vector. Seventy-two hours after electroporation, they demonstrated a noteworthy change in the mRNA levels of the *FGF2* and *VEGF* genes expression in modified cells by 19,000 ± 1186 and 57,000 ± 2250 times, respectively compared to unmodified UCB-MC^36^. Also, our previous study showed a significant enhancement in VEGF expression in fibroblasts cultured on the PU-CA/gelatin.PRGF/PU-CA scaffold^17^. Moreover, in different types of scaffolds, a significant increase in the VEGF expression has been reported in the primary bladder smooth muscle cells and bone marrow stem cells^37,38^.

Based on the results, the wound healing process of VEGF-Hu02 in the first 5 days was faster. However, wound healing with the same closure rate was represented on the fifteenth day in all different groups. Most studies^18,19,40^ affirmed that the PU scaffold could adhere uniformly to the wound surface without accumulating wound exudates. At the same time, PU in combination with other polymeric materials such as PU/EEP^39^, PU/EEP-PCL/Gel^39^, and PU/30WEP^40^ showed a better contraction percentage. In another study, Esmaeili et al. also reported adding other materials to PU-CA polymers, such as curcumin, reduced graphene oxide, and silver which showed a fast healing rate^18^.

Histopathology studies demonstrated appropriate effects of the VEGF-Hu02 on angiogenesis activity in the rat full-thickness wound skin model with PU-CA scaffold. Liao et al. investigated the effects of BMP‑6 and VEGF on BMSCs by transfection. They showed the combination of BMSCs with a biomimetic synthetic scaffold poly lactide‑co‑glycolide (PLAGA) in nude mice. After four weeks, histological analysis of the implants showed the most significant number of tubes, the most muscular capillary integrity, and bone formation in the AAV‑VEGF‑BMP‑6 group was remarkably higher than in the untransfected group^29^.

A representative histomorphometric analysis of collagen fiber distribution in the VEGF-Hu02 group was consistent with the histopathological observations. Since angiogenesis formation was more remarkable and faster in the manipulated group, more accumulation of collagen deposition was also detected. Accumulating collagen fibers with a parallel arrangement in the ECM of the VEGF-Hu02 group represented an acceleration of wound healing activity. Furthermore, most histological results from wound healing studies reported accelerated dermis development by wound dressing that resulted in complete wound closure two weeks post-surgery^39,41^. Although significant and early angiogenesis happened in manipulated fibroblasts during wound healing, wound closure was finally observed in all groups on day 15. Given the functional features of the VEGF-Hu02 group in the present study, these functions in larger-scale wounds will be significant. CD31 immunostaining also showed that the VEGF-Hu02 group improved the healing process by forming new vessels early and decreasing wound closure time. This result was in coordination with the findings of Spanholtz et al., who demonstrated an amelioration in skin flap survival. They showed enhancing the number of smooth muscle alpha-actin+/CD31+ blood vessels and decreased necrosis by 25% through transfection of *bFGF* and *VEGF*_*165*_ genes in fibroblast cells^42^. Salafutdinov et al. showed that transplantation of the hematopoietic stem cells expressing pro-angiogenic growth factors provides the higher flap revascularization processes in wounded skin. Moreover, endothelial markers, such as CD31 and CD144, two weeks post-transplantation in the tissues following skin wounds in rats revealed regenerative abilities. It was noted that these markers are expressed in endotheliocytes, hair follicle cells, fibroblast cells, and some mononuclear cells^36^.

## Conclusions

Our results suggest overexpressing VEGF in fibroblast cells enhances angiogenesis in vitro and in vivo. Fibroblast cells expressing VEGF are accountable for enhanced numbers of blood vessels and accelerating angiogenesis in the wound healing process, presenting theoretical and functional support for its use in gene therapy. We also concluded that applying pcDNA3.1 (-)-VEGF_165_ plasmid is efficient for the direct gene therapy of skin wounds by stimulating wound revascularization. Moreover, PU, as a nontoxic, biocompatible, and synthetic polymer, preppers a scaffold with cell affinity and progressed bioactivity for skin tissue regeneration when combined with CA. Animal studies also showed that accompanying the PU-CA scaffold with manipulated fibroblast cells can remarkably accelerate wound healing and shorten wound closure time. In conclusion, therapy with VEGF-overexpressing fibroblast cells significantly represented accelerating angiogenesis in the early stage, enhanced connective tissue, and improved wound healing.

## Methods

### Construction of expression vector

The Human *VEGF*_*165*_ gene (GeneBank AB021221.1) was cloned into pcDNA3.1/Hygro (-) (Thermo Fisher Scientific, USA) to construct pcDNA3.1(-)-VEGF. First, the 576 bp VEGF-coding sequence was obtained from the HepG2 cell line by reverse transcriptase-polymerase chain reaction (RT-PCR). For this purpose, PCR primers were designed as follows, where a polyhistidine tag (His-Tag) was incorporated in the reverse primer:

VEGF-F: 5′-ACATCAGGTACCATGAACTTTCTGCTGTCTTGG -3′, and

VEGF-R: 5′-ACATCTGGTACCAGCGGCCGCATGATGATGATGATGATGCCGCCTCGG

CTTGTCAC -3′.

The resulting *VEGF*_*165*_ fragment was amplified and cloned in the TG19-T intermediate vector (Vivantis, Malaysia). In the next step, the *VEGF*_*165*_ fragment from the intermediate vector was cloned downstream of the CMV promoter in the *BamHI* restriction site of the pcDNA3.1 (-) vector. All of the cloning steps were verified using restriction digestion analysis. The VEGF_165_ coding sequence was further confirmed using sequencing.

### Transfection and development of stable fibroblast cells containing VEGF_165_

1 × 10^5^ Hu02, human foreskin fibroblast cells that were purchased from the Iranian Biological Resource Center, cultured in 24-well plates one day before transfection in dulbecco’s modified eagle medium (DMEM)/F-12 medium supplemented with 2 mM L-glutamine and 10% fetal bovine serum (FBS) (Gibco, USA). Based on the manufacturer's instructions, the transfection was performed applying TurboFect reagent (Thermo Fisher Scientific, USA). Hu02 cells were transfected with 1 µg of the pcDNA3.1(-)-VEGF expression vector in duplicates. The pEGFP-puro reporter vector was also utilized as a control vector in the transfection procedure. Seventy-two hours after transfection, EGFP expression was assessed via fluorescence microscopy (Nikon Instruments, USA). The transfected cells were then mixed and transferred to 6-well plates. The pools were selected with hygromycin (200 µg/ml) (Invitrogen, USA) for 3 to 4 weeks.

### RNA extraction and real-time PCR

Quantitative real-time PCR (qRT-PCR) was investigated to analyze the expression levels of VEGF in stable pools and control cells (Hu02). Cells were cultured at the same density, and after 72 h, overexpression of VEGF was analyzed. Total RNA was extracted by TRIzol reagent (Invitrogen), according to the manufacturer's instructions. Then, the quantity of RNA was determined by NanoDrop (Aosheng, China) and 1 µg of total RNA was transcribed to complementary DNA (cDNA) through 1 µL of random hexamer primer. The reactions were incubated at 70 °C for 5 min. After that, 5X RT-buffer, dNTP, and RT-enzyme were added, and the mixtures were incubated at 42 °C for 60 min and 70 °C for 10 min. qRT-PCR was performed using the StepOnePlus system (Applied Biosystems, USA) with a high ROX SYBR Green master mix (Ampliqon, Denmark). The amplification program was adjusted at 95 °C for 15 min, followed by 40 cycles at 95 °C for 20 s and 60 °C for 1 min. The relative expression of the target gene in the experimental and control group was analyzed by the 2^–ΔΔCt^ method, *β-actin* housekeeping gene was used to normalize the relative expression. Table 1 provides the sequences of the primers used in this study^17^.

**Table 1:** Designed primers used in qRT-PCR.

Gene	Primer sequences 5′–3′
VEGF_165_	F: CCCATGGCAGAAGGAGGAG
	R: GGATGGCTTGAAGATGTACTCG
SGPL1	F:CATCTACCGACTATCAAACCTG
	R:GCGTGTAGTAATGTGTATGCAG
DDR2	F: GAGACCAAGGGAGGCAGAC
	R: CATCACTCGTCGCCTTGTTG
β-Actin	F: TCCTCCTGAGCGCAAGTAC
	R: CCTGCTTGCTGATCCACATCT

### Western blot

The specimens were lysed in radio immunoprecipitation assay buffer (RIPA buffer) (Cyto matin gene, Iran) with a phenyl methyl sulfonyl fluoride (PMSF) protease inhibitor (Sigma, USA) and centrifuged (15,000 rpm, 15 min, 4 °C). To assess protein concentration, the supernatant was collected and tested by the BCA assay kit. Protein samples (35–40 µg) were resolved by 12% sodium dodecyl sulfate–polyacrylamide gel electrophoresis (SDS-PAGE) (Bio-Rad, USA) for about 3 h at 95 V. Proteins were transferred from the gel into a nitrocellulose membrane (Amersham, UK) to perform the western blotting by applying the Semi-Dry-Blotting system (Bio-Rad, USA) for 45 min at 25 V. Nonspecific sites were blocked in 5% Skimmed milk (Sigma, USA) in Tris-buffered saline solution with tween 20 (TBST) for 1 h. The membrane was cut prior to hybridization with His-Tag antibody according to the ladder size. Following washing in tris-buffered saline (TBS) containing 0.1% tween 20, the membrane was incubated with a primary antibody against His-Tag (1 µg/mL, Novagen) overnight at 4 °C. Then, the membrane was rinsed three times with TBST and incubated with horse radish peroxidase (HRP) conjugated anti-human secondary antibody for 1 h at room temperature. Radiographic films and enhanced chemiluminescence (ECL) reagent (Amersham Pharmacia Biotech, Buckinghamshire, UK) were used for visualizing protein bands.

### Fabrication of scaffold

In order to fabricate the PU-CA scaffold, PU (Sigma Aldrich, USA) solution contained 4 wt% and CA (Sigma Aldrich, USA) with 18 wt% were prepared. PU was liquated in the 3:1 ratio of N, N dimethylformamide (DMF) (Merck, Germany) and tetrahydrofuran (THF) (Merck, Germany) for 15 h at room temperature on a stirrer to obtain a homogeneous solution. CA was also dissolved in acetic acid and H_2_O solutions in a ratio of 1:3 for 15 h at room temperature on a stirrer. After the desired time, PU and CA solutions were mixed in 3:1 ratio. Then, the uniform solution was run in an electrospinning device (Nano Spinner, Iran) under 16 kV voltage throughout in 18 cm distance between the nozzle tip and collector. The solvent evaporated and a collector gathered the scaffold fibers with a rotating drum speed of 300 rpm with a feeding rate of 0.2 mL/h. The PU-CA solution was electrospun for 8 h under the mentioned conditions.

### Characterization of electrospun scaffold

The morphology and structure of the synthesized scaffold were examined by scanning electron microscope (SEM). The gold layer was covered on the surface of the scaffold under 25 kV accelerated voltas and observed via Philips/FEI XL30FESEM (Philips, Netherlands). To determine the fiber diameter, the images acquired by SEM microscopy were analyzed by Image J software (National Institute of Health, USA). Mechanical characteristics of the synthesized scaffold were determined via tensile measurement on an Instron universal testing machine (Model STM‐20, SANTAM, Iran) at 5 mm/min speed with 50 newtons force. The scaffold was cut in 0.5 cm by 3 cm dimensions, firmed on paper frames, and exposed to tensile force for rupturing. The elastic modulus, strain at break, and final tensile strength were evaluated. Finally, the stress–strain response of the scaffold was plotted as a stress–strain curve. To identify molecular components, functional groups, and bonding configurations of the scaffold, the Fourier Transform Infrared Spectroscopy (FTIR) was used in the wavenumber range of 4000–400 cm^−1^.

Plasma cleaner was used for 3 min to enhance the surface hydrophilicity of the scaffold. Then, the contact angle was used to assess the surface hydrophilicity of the scaffold. To investigate a contact angle goniometer (Krüss, Hamburg, Germany), the scaffold was cut 1 × 1 cm, fixed on a support base, 3 µL of the DW was dropped on the surface, and then had been taken photos in less than 1 min.

### Cell adhesion analysis

The attachment of fibroblast cells was assessed through the 4,6-Diamidino 2-phenylindole (DAPI) (Sigma-Aldrich, Germany) staining test and SEM images. Briefly, 1 × 10^4^ cells/well were cultured on the PU-CA scaffold and DAPI staining was done after 7 and 14 days. In this way, cells were washed and incubated with 4% glutaraldehyde for 40 min and washed with PBS three times. DAPI stain was utilized to stain the cells’ nuclei in a dark place for 5 min. The specimens were rinsed with PBS and kept in the dark and cold places before being photographed by a fluorescent microscope (Nikon Instruments, USA). SEM images were taken 72 h after cell culture. First, the cultured cells on the scaffold were rinsed with PBS and fixed with glutaraldehyde (2.5%) for 2 h. Second, a serial dilution of ethanol (50–100%) was used to dehydrate the sample. Ultimately, the surface of the scaffold was coated with the gold layer, and Philips SEM took the images.

### MTT assay

The viability rate of cells on the PU-CA scaffold was measured using MTT (3-(4,5-dimethylthiazol-2-yl)-2,5-diphenyl tetrazolium bromide; Sigma, Germany) assay. 5 × 10^3^ fibroblast cells/wells were cultured on the scaffold in 48-well plates and 200 µL (DMEM)/F-12 culture media, containing 1% penicillin/streptomycin and 10% FBS, was added to each well. MTT assay was done on days 1, 3, 7 and 14. As a control group, tissue culture polystyrene (TCPS) was used in the mentioned conditions for the sample without a scaffold. After the desired time, the media was removed from the cells and the scaffolds were adjacent to the culture medium containing 10% MTT solution for 3 h in the incubator at 37 °C. Then dimethyl sulfoxide (DMSO) (Sigma-Aldrich, UK) dissolved formazan crystals. Finally, the optical density of the collected solution was recorded at 570 nm using a microplate reader (BioTek, USA).

### Gene expression analysis of cells on the scaffold

The qRT-PCR was applied to assess the expression level of migration, proliferation, and angiogenesis markers in manipulated fibroblast cells compared to fibroblast cells on the PU-CA scaffold after 3 days of culture. Sphingosine-1-phosphate lyase (*SGPL1*), discoidin domain receptor tyrosine kinase 2 (*DDR2)*, and *VEGF* were evaluated as proliferation, migration, and angiogenesis markers. The primer sequences used are listed in Table 1^17^. Total RNA from different samples on scaffold and TCPS group were extracted by TRIzol reagent (Invitrogen) based on the manufacturer’s instructions. Then, cDNA synthesis and the amplification program were executed as described in Sect. 2.3.

### In vivo wound healing experiments

The in vivo experiments were conducted on 32 male adult Sprague Dawley rats (Weight 250–300 g) purchased from the Laboratory Animal Center of the Faculty of Veterinary Medicine (Tehran University, Iran). Animal procedures were approved and carried out in accordance with relevant guidelines and regulations by the Animal Ethics Committee of Shahid Beheshti University of Medical Sciences (Tehran, Iran). Before the operation, all rats were anesthetized with xylazine (5 mg/kg) and Ketamine (7 mg/kg) mixture by intraperitoneal injection. The full-thickness excisional wound model was created on the rats’ dorsal skin on either side from the midline using 8 mm punch biopsies after perfect shaving and disinfection of the target region. Then, the wounded rats were randomly distinguished into four groups as follows: (1) wound only (blank control); (2) PU-CA scaffold; (3) PU-CA scaffold containing fibroblasts (PU-CA/Cell); and (4) PU-CAscaffold containing fibroblasts overexpressing VEGF (PU-CA/ Manipulated cells). The full-thickness skin excisions were developed for the wound region on days 0, 2, 5, 12 and 15. The scaffolds were used precisely on the wound site. An elastic adhesive bandage was utilized to sturdy and fix the dressings on the wounded region. Digimizer image analysis software (MedCalc Software bvba, Ostend, Bélgica) was used to evaluate wound healing progression by continuously measuring the wound diameters on days 0, 2, 5, 12 and 15. The wound’s pictures were taken over the certain days and the remaining wound areas’ percentage was measured according to the following equation:

1 $${\text{Remaining wound area percentage}} = \frac{{{\text{Wound area day 0}} - {\text{contracted wound area day}}\left( {\text{n}} \right)}}{{{\text{wound area day 0}}}} \times 100$$

### Histopathology study

Animals on mentioned days were sacrificed by ketamine overdose injection, and the collected tissues were placed in a 10% formalin solution and fixed for 24 h. After that, the samples were put in paraffin blocks, sectioned into 5 µm thick slices using a microtome (Leica Biosystems, Germany), and fixed on coverslips for future staining. The tissue sections were individually stained by Masson’s trichrome (MT) and Hematoxylin and Eosin (H&E) methods. The slides were dehydrated, mounted, and histological photographs were observed and interpreted by a digital light microscope (Olympus, Japan) at ×100 and ×200 magnification. In comparison, angiogenesis, inflammatory cell infiltration, reepithelialization, fibroproliferation, hemorrhage, connective tissue formation, and collagen deposition were assessed comparatively in different groups and on certain days. Histomorphometry analysis was conducted to evaluate the angiogenesis in the healing process.

### Immunohistochemistry (IHC) study

CD31, as a marker of angiogenesis, was used in different samples on the second day to identify newly formed blood vessels. A series of adjacent serial sections were cut from each sample and stained with a CD31 marker to evaluate the percentage of vascular lumen formation. For this purpose, blocked specimens in paraffin were sliced into 5‐µM thick sections, deparaffinized with xylene, and rehydrated with reducing concentrations of ethanol in water. A microwave oven performed heat-induced epitope retrieval. The slides were incubated with 3% hydrogen peroxide for internal peroxidase blocking for 20 min. Primary antibody (clone JC/70A, mouse monoclonal, IgG1/kappa, Ready to use, catalog PDM020-10MM, Diagnostic Biosystems, The Netherlands), and diaminobenzidine tetrahydrochloride (DAB) solution were applied.

### Statistical analysis

Data were expressed as the mean ± standard deviation (SD). Two-way ANOVA was employed to compare the mean differences among multiple groups that have been divided into two independent variables. The student’s *t*-test was also applied to compare the two groups. *P* values < 0.05 were evaluated to be statistically significant in all valuations.

### Ethical approval

Our experimental protocol for animal study are performed according to the guidelines approved by Shahid Beheshti University of Medical Sciences. The current project has succeeded in receiving the code of ethics with the number (IR.SBMU.RETECH.REC.1397.244) from Shahid Beheshti University of Medical Sciences. All the ethical issues have been monitored during the project to decrease the animals suffering in accordance with the existing protocols. This study was reported by ARRIVE guidelines (https://arriveguidelines.org).

## Supplementary Information

Supplementary Information.

## Data Availability

The dataset generated and analyzed during the current study is available in the Mendeley Data, https://data.mendeley.com/datasets/pxhhfrbs64/1.
