# Supplementary material for: Overexpression of VEGF in dermal fibroblast cells accelerates the angiogenesis and wound healing function: in vitro and in vivo studies
Source: Sci Rep. 2022 Nov 2;12:18529. doi: 10.1038/s41598-022-23304-8 (PMC9630276; doi:10.1038/s41598-022-23304-8)
Supplement: Supplementary file 1 — Supplementary Information. [file 41598_2022_23304_MOESM1_ESM.docx]

This file includes:

Supplementary Figures 1-2


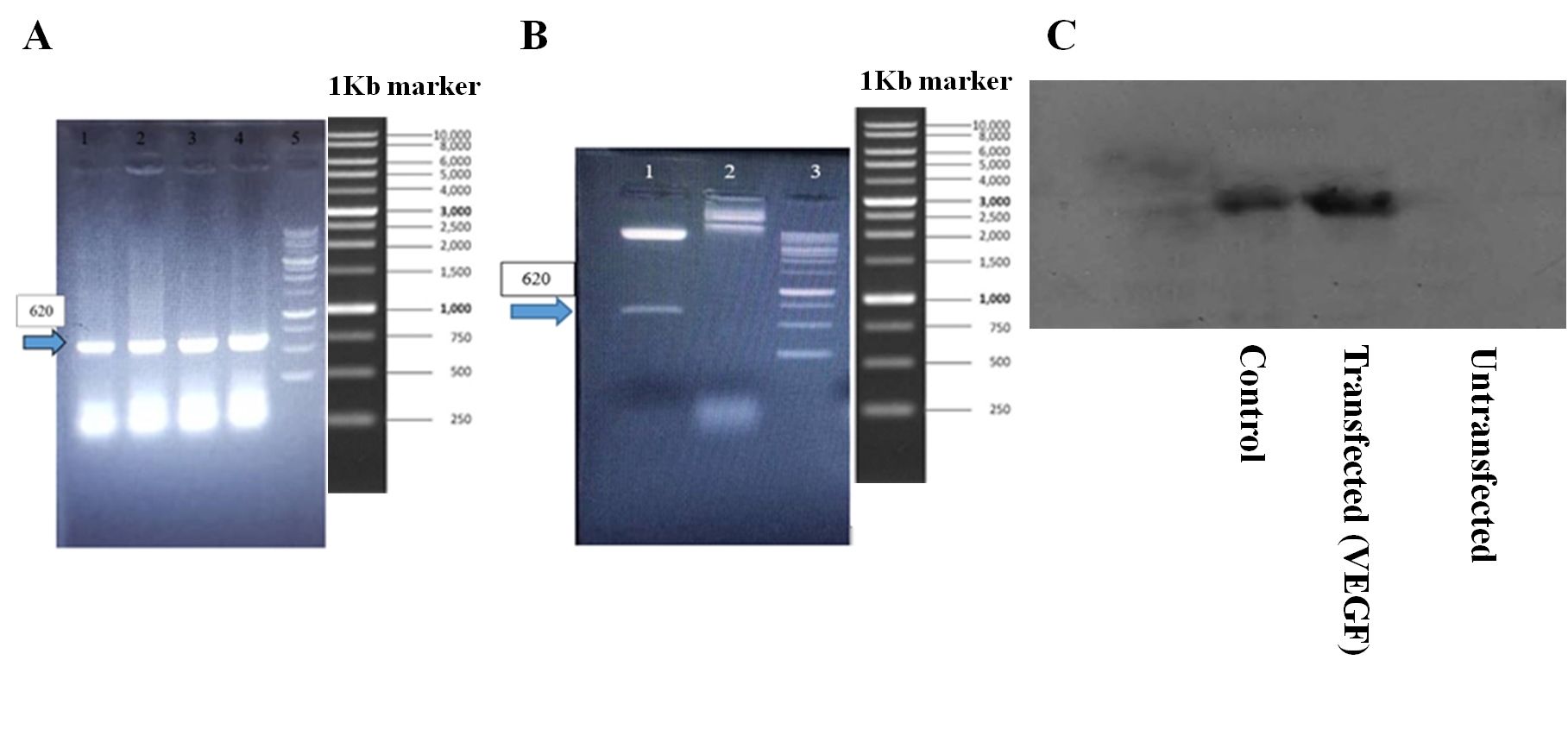
 Supplementary Figure 1. (A) The original and unprocessed version of figure.1B (Uncropped). (B) The original version of figure.1C. (C) The unprocessed version of the figure.2C

Supplementary Figure 2. Presentation of Figures 7 on days 2 (A1-A4) at higher magnification. A1.1 (Control) and A2.1 (Scaffold): Tissue necrosis with high neutrophil infiltration and no angiogenesis. A3.1 (Scaffold+Hu02): Few formations of new blood vessels (yellow arrow). A4.1 (Manipulated Hu02): Remarkable angiogenesis (yellow arrow).
